# Challenges and Considerations in Optimizing Ovarian Stimulation Protocols in Oncofertility Patients

**DOI:** 10.3389/fpubh.2014.00246

**Published:** 2014-12-05

**Authors:** Kathryn Coyne, MacKenzie Purdy, Kathleen O’Leary, Jerome L. Yaklic, Steven R. Lindheim, Leslie A. Appiah

**Affiliations:** ^1^Department of Obstetrics and Gynecology, Boonshoft School of Medicine, Wright State University, Dayton, OH, USA; ^2^Department of Obstetrics and Gynecology, University of Kentucky College of Medicine, Lexington, KY, USA; ^3^Wright-Patterson USAF Medical Center, Dayton, OH, USA

**Keywords:** oncofertility, controlled ovarian stimulation, assisted reproduction, follicular phase, luteal phase

## Abstract

The scope of cancer treatment in women of childbearing age has changed in the last decade. Fertility preservation is no longer an afterthought but central to multi-disciplinary cancer treatment planning and should be addressed due to the cytotoxic effects of cancer therapy. However, oncology patients present as a unique treatment challenge as the physician must balance the urgency of fertility preservation with the risks of delaying cancer therapy. Controlled ovarian stimulation (COS) is routinely applied in assisted reproductive technology but can be contraindicated in women with estrogen-receptor-positive tumors. This paper reviews some of the challenges to consider when using COS and newer stimulation protocols to minimize risks and optimize outcomes in oncofertility patients.

## Introduction

Fertility preservation for female cancer patients prior to cancer therapy has emerged as an essential component of comprehensive patient care. In a survey questioning, the importance of fertility preservation in women of childbearing age recently diagnosed with cancer, 77.6% (66 of 85) reported that the possibility of preserving their fertility was instrumental to coping before and after treatment (1).

In the U.S., approximately 6% of women diagnosed with invasive cancer between 2007 and 2011 were <45 years old (2). The incidence of cancers in reproductive age women signifies a need for fertility preservation options, and this need is increasing with the current trend of delayed childbearing and increased cancer survival. The birth rate for women aged 35–44 rose 54% from 1990 to 2011, increasing the number of women diagnosed with cancer who may wish to conceive after cancer treatment (3). Additionally, there has been a significant increase in cancer survival rates during the past decade due to earlier diagnoses and improved cancer treatments (4). From 2002 to 2012, 83% of female patients <45 years old diagnosed with cancer survived >5 years (5).

Cancer treatment is cytotoxic and may result in complete or partial ovarian failure with subsequent subfertility, sterility, and premature menopause. All chemotherapies have the potential to damage developing follicles; this can result in temporary amenorrhea. If the primordial follicle pool is not permanently damaged, menses will return, with the development of new follicles within 3–6 months after the last treatment (6). Studies indicate that alkylating agents are particularly gonadotoxic (7), with significantly diminished ovarian response demonstrated in those who received alkylating agents prior to oocyte retrieval (8). Pelvic radiation therapy is also highly gonadotoxic to oocytes, causing follicular destruction, resulting in premature ovarian insufficiency in many women (9). Similar to chemotherapy, this follicular depletion and ovarian decline from treatment accelerates the onset of menopause (10). The resulting damage is dose-related and depends on ovarian reserve and age at time of treatment (11). Young women are presumed to be less affected by gonadotoxic agents due to a larger reserve of primordial follicles, but it is established that they are at risk of accelerated follicular loss, premature ovarian failure, and infertility (12).

Effects of cancer may also include an increased catabolic state, stress, and malnutrition, resulting in an adverse effect on all organ systems. Furthermore, enhanced stress hormones and hypothalamic dysfunction lead to decreased levels of gonadotropins, which in turn impacts fertility (13). These factors may lead to diminished ovarian reserve, yielding lower oocyte recovery at oocyte retrieval (14). For breast cancer patients, oocytes may be at risk for DNA damage, resulting from BRCA gene mutations (15). A meta-analysis demonstrated a statistical significance when comparing the number of retrieved oocytes for those in the cancer group compared to controls: 11.7 ± 7.5 vs. 13.5 ± 8.4, *p* = 0.002 (95% CI, −2.976; −0.621) (14). Retrievals and cancelations were significantly lower and higher, respectively, in cancer patients compared to healthy age-matched individuals (14). Thus, the ovarian reserve and oocyte quality of cancer patients prior to undergoing fertility preservation may already be poorer than that of a healthy individual.

Multiple strategies are available to preserve fertility in these patients, including embryo and oocyte cryopreservation, cortical and whole ovary cryopreservation, ovarian transplantation, ovarian transposition, *in vitro* maturation of immature oocytes, and ovarian suppression with gonadotropin-releasing hormone (GnRH) analogs (16). There have been several recent advancements in assisted reproductive technology (ART) that provide potential new options for fertility preservation in this patient population. Currently, embryo and mature oocyte cryopreservation following *in vitro* fertilization (IVF) are the only techniques endorsed by the American Society for Reproductive Medicine (ASRM) as standard therapies; all other methods are still considered investigational (17, 18).

Controlled ovarian stimulation (COS) for oocyte/embryo cryopreservation is still the preferred method for fertility preservation due to its higher success compared to other technologies. Special considerations must be taken into account for a cancer patient undergoing fertility preservation using COS, including the optimal dosing, timing strategies, and the risks of increased estrogen exposure and delay in treatment with those who develop ovarian hyperstimulation syndrome (OHSS). This paper will review the current knowledge of fertility preservation options and the clinical challenges and strategies to optimize treatment outcomes in cancer patients undergoing fertility preservation.

## Challenges and Considerations

### Time constraints and avoiding risks

Preserving a woman’s fertility requires time for ovarian stimulation and oocyte retrieval. Traditionally, COS is initiated at the start of the follicular phase with the premise that it is the optimal time for recruitment of the ovarian follicular pool, maximizing the number of retrieved oocytes. This is particularly important as there may be time for only one cycle of COS prior to initiating cancer therapy. However, waiting for the patient’s menstrual cycle may require several weeks until one can undergo COS, which would delay life-saving cancer therapy.

Ovarian hyperstimulation syndrome, an iatrogenic sequelae of COS, is the most serious complication of ovarian stimulation, occurring in 3–8% of IVF cycles (19), and cancer patients risk a delay in therapy if OHSS develops (20). OHSS, in its severest form, is associated with intravascular depletion, ascites, liver dysfunction, pulmonary edema, electrolyte imbalance, and thromboembolic events. It is usually self-limited with spontaneous resolution in a few days, but may progress in severity, rarely requiring hospitalization.

Thromboembolic events are one of the most concerning events as patients with a neoplasm inherently have a hypercoagulable state that poses an increased risk of morbidity and mortality (21). Cancer patients may therefore be at even greater risk if OHSS develops following COS. Thus, identifying the optimal COS strategy to maximize oocyte recruitment while preventing OHSS is most ideal to avoid this serious complication.

### Concerns with estrogen-sensitive cancers

Elevated serum estradiol (E_2_) levels as a result of COS with gonadotropins may promote growth of tumors in estrogen-sensitive cancers, such as endometrial and estrogen-receptor-positive breast cancers (22). The rise in E_2_ levels is directly proportional to the number of recruited follicles, thus, protocols for these patients must aim to reduce estrogen production (23).

### Prepubescent and adolescent patients

Fertility preservation in pediatric and adolescent oncology patients encompasses the full range of standard and experimental options. In the prepubescent patient, ovarian tissue cryopreservation is the only option and is still investigational. In the adolescent patient, egg and embryo freezing are standard options similar to reproductive age women while ovarian tissue freezing remains investigational.

Some of the challenges include the process of patient assent and parental consent and a thorough understanding of the process of daily injections, serial ultrasounds, and lab testing with the challenges of cost, time, discomfort, and posthumous related issues. These challenges can be mitigated by a comprehensive and realistic discussion of the process using a team approach of empathic nurses, social workers, and a financial team. Embryo freezing may not be a feasible option in the adolescent patient who may not be able to consent to use of partner or donor sperm. If time is limited, ovarian tissue cryopreservation is an option. It involves an oophorectomy typically with a minimally invasive approach often combined with central line or port placement for chemotherapy, thereby minimizing anesthetic risk and cost. As ovarian tissue freezing remains experimental with 24 babies reported, to date (24), establishing ovarian stimulation protocols for egg freezing that maximize outcome and minimize discomfort in the adolescent patient are necessary. *In vitro* maturation of immature oocytes is a promising investigational technique that would negate the time required for ovarian stimulation and would be an option for both pre- and post-pubescent patients (25).

### Assessment to optimize COS

The efficacy of oocyte retrieval and ovarian stimulation is directly related to the quantity and quality of the patient’s current ovarian reserve. Therefore, assessing reserve is important in counseling the patient adequately and to utilize protocols to optimize oocyte yield while minimizing development of OHSS. Ovarian reserve tests include basal follicle-stimulating hormone (FSH), inhibin B, antral follicle count (AFC), ovarian volume assessed by transvaginal ultrasound, and anti-Mullerian hormone (AMH). Unlike basal cycle day 2–3 FSH and estradiol, serum levels of AMH, which can be performed at any time of the menstrual cycle, and AFC serve as good predictors of ovarian reserve and response to COS. With respect to those with cancer, there are mixed reports on the predictive value of AMH (26) and AFC (27) compared to patients without a cancer diagnosis. Responses to COS are significantly lower in cancer patients with lower AMH and AFC prior to undergoing chemotherapy with the requirement of higher doses of gonadotropins compared to healthy age-matched women (28). It is therefore likely that the response to COS is related to both the patient’s current ovarian reserve and overall clinical condition (29). In addition, AMH neither predict spontaneous conception nor pregnancy or live birth after IVF (30). As such, these factors are not definitive in predicting outcomes but are useful for counseling patients, helping to develop realistic expectations, and determining a safe and effective ovarian stimulation protocol for the cancer patient undergoing fertility preservation (31).

## Current and Potential Ovarian Stimulation Protocols

### Conventional controlled ovarian stimulation protocols

**Figure 1:**
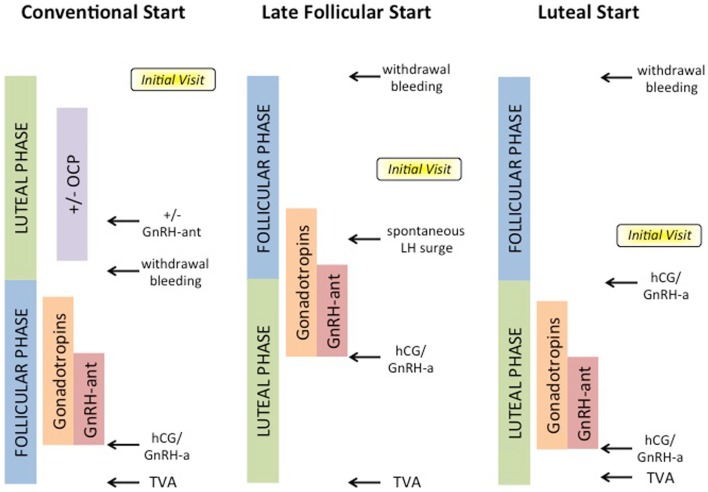
**Options for COS for ART cycles**. Conventional-start begins in the luteal phase with or without administration of oral contraceptive pills (OCPs) or GnRH-antagonists (GnRH-ant), and proceeds after withdrawal bleeding with gonadotropins and GnRH-ant, until hCG or GnRH-a trigger, and lastly transvaginal aspiration (TVA) of oocytes. Random-start protocols may begin either in late follicular or luteal phases, and may utilize spontaneous LH surge or GnRH-a.

Conventional-start COS is currently the preferred and established method of ovarian stimulation for cancer patients (29). This protocol is initiated at the beginning of the follicular phase, and requires 9–14 days of ovarian stimulation with gonadotropins followed by 14 days of ovarian suppression with a GnRH agonist (GnRH-a) during the luteal phase to prevent premature ovulation (Figure 1). Thus, depending on a woman’s cycle at time of diagnosis, conventional-start COS may require 4–6 weeks, which could represent a significant delay in commencing cancer therapy. COS and IVF would not be a viable option for patients with rapidly progressing neoplasms that require immediate therapy.

The development of newer generation GnRH-antagonists (GnRH-ant) have successfully prevented a premature luteinizing hormone (LH) surge during ovarian hyperstimulation for IVF cycles. This is more optimal than conventional GnRH-a, allowing the shortest delay of cancer therapy as it decreases the time between diagnosis and oocyte or embryo cryopreservation (32). In comparison to traditional GnRH-a, GnRH-ant has the advantage of immediate suppression of pituitary gonadotropins, preventing an FSH and LH flare. This obviates the prolonged period to pituitary suppression and has been reported to result in shorter treatment cycles and less gonadotropin requirements (33).

For cancer patients waiting for their menses, GnRH-ant have been administered during the preceding mid-luteal phase with menses occurring a few days later (34). An alternate strategy is to use oral contraceptive pills (OCPs) as pretreatment to regulate and control withdrawal bleeding (35). OCPs avoid the need to wait for natural onset of menses and deter selection of a lead follicle. They are only an option in non-estrogen-sensitive cancers.

### Random-start controlled ovarian stimulation protocols

Random-start COS protocols are emerging as an attractive approach as they minimize delays in cancer therapy (29, 36–38). Two different approaches, late follicular and luteal phase, have been suggested for random-start COS protocols (Figure 1).

#### Late follicular phase

Late follicular phase protocols begin after menstrual cycle day 7 with emergence of a dominant follicle (>13 mm) (36). If the follicle cohort following the lead follicle is <12 mm before a spontaneous LH surge, a protocol utilizing ovarian stimulation without a GnRH-ant may be employed (36). After the LH surge, a GnRH-ant is started later in the cycle when the secondary follicle cohort is 12 mm to prevent a premature secondary LH surge. If the follicle cohort following the lead follicle is >12 mm before a spontaneous LH surge, a protocol with a GnRH-ant for pituitary suppression may be initiated (36). This GnRH-ant is continued until final oocyte maturation with an hCG or GnRH-a trigger.

#### Luteal phase

Luteal phase protocols initiate ovarian stimulation in the absence of GnRH-ant. If the oncology patient presents in the luteal phase or ovulation was induced with hCG or GnRH-a, ovarian stimulation starts within 2–3 days in the early luteal phase (36). Similar to conventional-start COS-GnRH-ant protocols, these luteal phase start protocols utilize a GnRH-ant administered later in the cycle to prevent a premature LH surge, and continue until the hCG or GnRH-a triggers final oocyte maturation.

A study comparing stimulation in oncology patients with follicular to luteal phase starts showed similar average numbers of aspirated oocytes (13.1 vs. 10.0%) and fertilization rates (61.0 vs. 75.6%) (38). When comparing conventional-start COS and random-start COS, Cakmak et al. demonstrated that the numbers of total metaphase-II oocytes retrieved and fertilization rates were similar in oncology patients (36). While random-start COS resulted in longer length of ovarian stimulation by ~2 days (*p* < 0.001) and required higher average gonadotropin use (4,158 vs. 3,404 IU/day, *p* = 0.002), there was a significant advantage to random-start COS with respect to time to treatment for cancer (36). This is consistent with a novel concept that follicular recruitment occurs in multiple waves during each menstrual cycle (39).

### COS in patients with estrogen-sensitive cancer

Natural-cycle IVF without ovarian stimulation would be safe but only produces one or two oocytes per cycle, has a high-cycle cancelation rate, and thus, is not an optimal treatment choice even if there is little time before onset of cancer therapy (29). There are several alternative and safer protocols for estrogen-sensitive cancer patients that have been developed, though the efficacy of these various protocols has yet to be well established.

For patients with estrogen-sensitive cancers, protocols have been designed that include competitive inhibitors of estrogen receptors or aromatase inhibitors (AIs) to reduce estrogen’s effect or production, respectively. Tamoxifen is an estrogen agonist-antagonist with agonist effects at the uterus and bone and antagonist effects in the breast and central nervous system (CNS). Specifically, tamoxifen inhibits growth of breast tumors through its anti-estrogenic action and is used as first-line treatment and hormonal prevention in estrogen-sensitive cancers (40). In the CNS, it interferes with the negative feedback of estrogen on the hypothalamic-pituitary axis. This leads to increased GnRH secretion from the hypothalamus, FSH release from the pituitary, and stimulation of follicular development. In the uterus, tamoxifen has been found to cause hyperplasia and increase the risk of uterine cancer; therefore, it would not be recommended over AI for augmenting COS.

Tamoxifen can be used for COS alone on cycle days 2 through 5 of the patient’s menstrual cycle or in combination with gonadotropins (23). Ovarian stimulation with tamoxifen alone in cancer patients increases the mature oocyte and embryo yield compared with natural-cycle IVF and reduces cycle cancelations (41). With a combined treatment of tamoxifen and gonadotropins, this results in an even greater number of cryopreserved oocytes/embryos (42). This is especially important due to the fact that hormone-dependent cancers have been shown to have a weaker response to COS compared to non-hormone-dependent cancers (43).

Aromatase inhibitors, such as letrozole, reduce estrogen production. Aromatase catalyzes the conversion of androstenedione to estrone and of testosterone to E_2_ (44). AIs suppress plasma estrogen levels by competitively inhibiting the activity of aromatase (45), with the goal of keeping E_2_ levels close to those observed in natural cycles (46). In the CNS, AIs work similarly to estrogen inhibitors by blocking the negative feedback of estrogen on the hypothalamic-pituitary axis, resulting in FSH release from the pituitary and increased follicular growth (47). Letrozole is thus effective at preventing estrogen production and can also be used for ovulation induction.

Protocols with daily letrozole along with gonadotropins are preferred over tamoxifen because letrozole results in greater retrieved and fertilized oocytes (42). Short-term follow-up of breast cancer patients who had undergone ovarian stimulation with letrozole and gonadotropins has not revealed any increased risk of breast cancer recurrence (48). COS with AIs has also been safely used in endometrial cancer patients (49). As such, AIs are recommended as part of a safe, well tolerated, and effective protocol for fertility preservation.

### Minimizing risk of OHSS

During COS, it has been established that triggering final oocyte maturation with hCG may induce OHSS. Recent data suggest that GnRH-agonists (Leuprolide 0.5–1.0 mg SC) used to stimulate an endogenous LH surge for oocyte maturation results in reduced risk of OHSS due to the short half-life of the endogenously induced LH surge and lower estrogen production, which is believed in part to be the trigger for OHSS (50). When comparing the two, GnRH-a reduce the risk of OHSS in autologous IVF cycles. In fertility preservation cases, women with breast cancer also had significantly reduced risk of OHSS (2.1 vs. 14.4%, *p* = 0.032) by using GnRH-a triggers vs. hCG (51). However, this approach is only useful in cycles not involving previous down-regulation with GnRH-a, but may prove advantageous for patients with estrogen-sensitive cancers by decreasing the progression of their cancer. Future investigation is needed in this area due to limited studies determining cancer progression after GnRH-a triggering (52).

One potential concern with GnRH-a is that trigger failures may occur due to incomplete binding at GnRH receptors due to competition with GnRH-ant leading to a limited LH surge (53). Thus, in GnRH-ant fertility preservation protocols, a GnRH-a trigger is only recommended for those who are at risk for OHSS (29), and should be considered in the cancer patient who is to undergo chemo-radiation therapy upon completion of COS (52).

Withholding gonadotropin stimulation and delaying hCG administration until E_2_ levels plateau or decreases can reduce the risks of OHSS. In autologous IVF cycles, evidence suggests that “coasting” does not adversely affect outcomes in IVF cycles unless it is prolonged >3 days (54, 55). To date, no reports on this approach in fertility preservation and risks of OHSS have been reported (56).

### Possible prophylactic co-treatment

Oncology patients are at increased risk of thromboembolic events during COS because of their hypercoaguable state due to malignancy and supraphysiologic serum E_2_ levels due to stimulation (57). Anticoagulants, such as low-molecular-weight heparin, can be administered to prevent coagulation, and letrozole may be used to maintain low serum E_2_ levels in order to prevent this risk (29). In patients with bone marrow malignancies or liver involvement, it is important to be vigilant about excessive bleeding and recognize that platelet or fresh frozen plasma transfusion prior to oocyte retrieval may be required (29). Neutropenic patients are at risk of pelvic infection after retrieval; therefore, it may be appropriate to consult the oncologist with recommendations for antibiotics and/or granulocyte colony-stimulating factor to prevent infection.

In addition, ovarian suppression with GnRH-a during adjuvant chemotherapy may be an option for patients without time or resources available for other methods of fertility preservation (58). Currently, studies involving GnRH-a for prevention of ovarian failure after cytotoxic treatment are conflicting. However, this treatment is often used for menstrual suppression in oncology patients and thus a secondary benefit may be achieved with little risk of harm.

## Conclusion

Ovarian stimulation protocols must be individualized based on time available prior to cancer treatment and fertility status of the patient. Fertility preservation for oncology patients should be carried out with a multi-disciplinary approach, including oncologists and fertility specialists (59). Patients should meet with a reproductive endocrinologist as soon as possible after diagnosis of cancer in order to begin consultation and treatment (60).

The main goal of fertility preservation treatment for female cancer patients should be to maximize the number of oocytes/embryos preserved while avoiding OHSS and delayed cancer therapy. Conventional-start COS is an established method in fertility preservation and random-start COS is a promising method for retrieval of oocytes in urgent settings. AIs with gonadotropin are recommended in patients with estrogen-sensitive cancers and should be an essential component of a COS protocol for these patients. In all cases, ovarian reserve and E_2_ levels should be assessed prior to initiating COS to avoid adverse effects.

Other methods may emerge as more effective and safer in preserving fertility in urgent or estrogen-sensitive cases. *In vitro* maturation of oocytes is one such proposed method of fertility preservation that would be advantageous in these settings; however, at this time, it is still considered an experimental procedure (18, 61). Similarly, freezing of cortical strips of ovarian tissue is also a potential option although investigational. To date, few oncology patients have utilized their cryopreserved embryos and oocytes post-treatment, thus, data regarding efficacy in this population are lacking. While pregnancy outcomes in this population are still unknown overall, a previous study by Noyes et al. shows promising results (62). Further research and alternative protocols may be necessary and crucial in providing a greater opportunity for fertility preservation and better comprehensive care of female cancer patients.

## Conflict of Interest Statement

The authors declare that the research was conducted in the absence of any commercial or financial relationships that could be construed as a potential conflict of interest.
